# Demonstrating the benefits of corrective intraoperative feedback in improving the quality of duodenal hydrogel spacer placement

**DOI:** 10.1002/mp.15665

**Published:** 2022-04-18

**Authors:** Hamed Hooshangnejad, Sarah Han‐Oh, Eun Ji Shin, Amol Narang, Avani Dholakia Rao, Junghoon Lee, Todd McNutt, Chen Hu, John Wong, Kai Ding

**Affiliations:** ^1^ Department of Biomedical Engineering Johns Hopkins School of Medicine Baltimore Maryland USA; ^2^ Department of Radiation Oncology and Molecular Radiation Sciences Johns Hopkins School of Medicine Baltimore Maryland USA; ^3^ Carnegie Center for Surgical Innovation Johns Hopkins School of Medicine Baltimore Maryland USA; ^4^ Department of Gastroenterology Johns Hopkins School of Medicine Baltimore Maryland USA; ^5^ Division of Biostatistics and Bioinformatics Sidney Kimmel Comprehensive Cancer Center Johns Hopkins School of Medicine Baltimore Maryland USA

**Keywords:** FEMOSSA duodenal virtual spacer, spacer‐enabled pancreatic cancer radiotherapy, virtual spacer corrective feedback

## Abstract

**Purpose:**

Pancreatic cancer is the fourth leading cause of cancer‐related death with a 10% 5‐year overall survival rate (OS). Radiation therapy (RT) in addition to dose escalation improves the outcome by significantly increasing the OS at 2 and 3 years but is hindered by the toxicity of the duodenum. Our group showed that the insertion of hydrogel spacer reduces duodenal toxicity, but the complex anatomy and the demanding procedure make the benefits highly uncertain. Here, we investigated the feasibility of augmenting the workflow with intraoperative feedback to reduce the adverse effects of the uncertainties.

**Materials and Methods:**

We simulated three scenarios of the virtual spacer for four cadavers with two types of gross tumor volume (GTV) (small and large); first, the ideal injection; second, the nonideal injection that incorporates common spacer placement uncertainties; and third, the corrective injection that uses the simulation result from nonideal injection and is designed to compensate for the effect of uncertainties. We considered two common uncertainties: (1) “Narrowing” is defined as the injection of smaller spacer volume than planned. (2) “Missing part” is defined as failure to inject spacer in the ascending section of the duodenum. A total of 32 stereotactic body radiation therapy (SBRT) plans (33 Gy in 5 fractions) were designed, for four cadavers, two GTV sizes, and two types of uncertainties. The preinjection scenario for each case was compared with three scenarios of virtual spacer placement from the dosimetric and geometric points of view.

**Results:**

We found that the overlapping PTV space with the duodenum is an informative quantity for determining the effective location of the spacer. The ideal spacer distribution reduced the duodenal V33Gy for small and large GTV to less than 0.3 and 0.1cc, from an average of 3.3cc, and 1.2cc for the preinjection scenario. However, spacer placement uncertainties reduced the efficacy of the spacer in sparing the duodenum (duodenal V33Gy: 1.3 and 0.4cc). The separation between duodenum and GTV decreased by an average of 5.3 and 4.6 mm. The corrective feedback can effectively bring back the expected benefits from the ideal location of the spacer (averaged V33Gy of 0.4 and 0.1cc).

**Conclusions:**

An informative feedback metric was introduced and used to mitigate the effect of spacer placement uncertainties and maximize the benefits of the EUS‐guided procedure.

**Abbreviations**
CT: Computed tomography
EUS: Endoscopic ultrasound
GTV: gross tumor volume
HOP: head of pancreas
OAR: organs at risk
OS: overall survival rate
OVH: overlapped volume histogram
PTV: Planning Target Volume
RT: radiation therapy
SBRT: stereotactic body radiation therapy

## 1 INTRODUCTION

Pancreatic cancer is the fourth leading cause of cancer‐related death and the 12th most common malignancy in the US, with less than a 10% 5‐year overall survival rate (OS).^1^ Radiation therapy (RT) together with dose escalation increases the OS at 2 years from 19 to 36%, and at 3 years from 9 to 31%.^2^ However, the effectiveness of dose escalation is limited due to the proximity of organs at risk (OAR), mainly the duodenum. Our group has shown that injection of hydrogel spacer between the head of pancreas (HOP) and duodenum increases the duodenal sparing, and therefore, makes the dose escalation more feasible.^3^, ^4^, ^5^, ^6^, ^7^, ^8^, ^9^, ^10^, ^11^, ^12^, ^13^, ^14^, ^15^, ^16^, ^17^

However, the success of the spacer placement procedure is highly uncertain. Previous studies on rectal spacer have shown that hydrogel spacer injection is associated with risk of infection, inflammation, and soft‐tissue wall infiltration.^18^, ^19^, ^20^, ^21^ Moreover, misplacement of the spacer may result in patient discomfort and reduce the effectiveness of the spacer for OAR sparing.^20^, ^22^ The same risks and uncertainties may affect the duodenal hydrogel spacer placement. In addition, the duodenal spacer placement is considerably more complicated than rectal spacer placement. Due to the hard‐to‐reach location of the duodenum‐pancreas interface, the spacer is injected through an endoscopic ultrasound (EUS)‐guided procedure.

We hypothesize that a novel spacer placement workflow for duodenal hydrogel spacer featuring corrective intraoperative feedback will increase the robustness of minimally invasive EUS‐guided procedure, and will reduce the associated risks and uncertainties. Thus, the purpose of this study was to find the most informative feedback to guide the spacer injection procedure, and second, to show the feasibility and benefit of using the corrective feedback and injections to optimize the placement of spacer. We believe that the intraoperative feedback and corrective injection increase the efficiency of delivering the preoperative ideal spacer placement plan and, thus, the entire procedure.

First, the article explains how the data were collected and prepared for the study, and then describes the method used to simulate common uncertainties of spacer insertion. Next, it provides information on the radiotherapy planning protocol, and finally, focuses on introducing the informative feedback for corrective injection and evaluating the result from various aspects.

## 2 MATERIAL AND METHODS

### 2.1 Data collection and preparation

For this study, we used the data from four cadavers injected with hydrogel spacer. For each cadaver, two computed tomography (CT) scans are available, before hydrogel spacer placement and after the injection of hydrogel spacer. A biodegradable polyethylene glycol hydrogel (TraceIT, Boston Scientific, Bedford, MA) was injected through 18‐gauge needle under EUS guidance in the pancreaticoduodenal groove. This allowed us to, first, validate our spacer simulation algorithm on paired pre‐, postinjection scans, and then, use the platform to perform the spacer simulation study on preinjection scans with high confidence. All ROIs (Region of Interest) were segmented by a certified physician in our institute. All scans were acquired with 3‐mm slice thickness, 120 kVp, 200 mA, and field of view of 50 cm. For further analysis, CT scans and contours were exported as Digital Imaging and Communications in Medicine using commercial software, Varian Velocity. The anonymized data were then imported to MATLAB for simulation and analysis.

### 2.2 Finite element model‐oriented spacer simulation platform (FEMOSSA)

We simulated the duodenal spacer placement scenarios using our in‐house finite element‐based spacer simulation platform, finite element model‐oriented spacer simulation (FEMOSSA).^23^, ^24^ Here, we summarize how FEMOSSA performs patient‐specific spacer simulation. The 3D binary organ contours were imported and used to create a triangular surface mesh. A volume‐preserving Laplacian smoothing algorithm was applied to the triangular surface mesh to create a more realistic organ shape. Next, a 3D four‐node tetrahedral volume mesh bounded to the triangular surface mesh was generated and modified to create an accurate representation of duodenum wall. An in‐house algorithm generated a volume 3D mesh for the duodenum wall bounded to two triangular surface meshes 2 to 3 mm away, based on previous measurements.^25^, ^26^, ^27^

The HOP was modeled with a linear elastic behavior as a homogeneous, incompressible isotropic material, with Young's modulus of 30 kPa, and a Poisson coefficient of 0.48.^28^, ^29^, ^30^ The duodenum, however, due to its hollow structure, has a nonlinear, hyperplastic behavior.^31^, ^32^ As a result, an exponential Fung‐type strain energy function (Equation 1) was chosen in the commercial finite‐element analysis software package, ABAQUS (Version 6.4, ABAQUS Inc, Pawtucket, RI), for duodenum as suggested by the previous study^32^:

(1) $$\begin{array}{ccc} W\left(Q\right) & = & \frac{1}{2}c\left(e^{Q}-1\right)\;and\;Q\left(E\right)\\ & & =a_{1}E_{11}^{2}+a_{2}E_{22}^{2}+2a_{3}E_{11}E_{22}, \end{array}$$

where $E_{ii}$ indicates the strain, and *c*, *a*_1_, *a*_2,_ and *a*_3_ parameters were 1.05, 41.4, 51.1, 13.2, respectively.

The spacer injection process was defined as a translation of an ensemble of blebs from an initial position, toward the final position, the desired spacer distribution. To initialize the simulation of bleb‐surface contact, each bleb was placed tangent to the contour surface that is going to be deformed, as close as possible to its final location. The desired spacer distribution, the final position of blebs, was created by placing an ensemble of spherical objects (blebs) with various radii using the FEMOSSA built‐in graphical user interface. The blebs push the proximal contour surface on their way from the initial to the final position, and thus, deform organs during this transition. This innovative and simplified definition was used to turn this complex physical phenomenon into a manageable quasi‐static problem while capturing the dynamics of the process.

**FIGURE 1:**
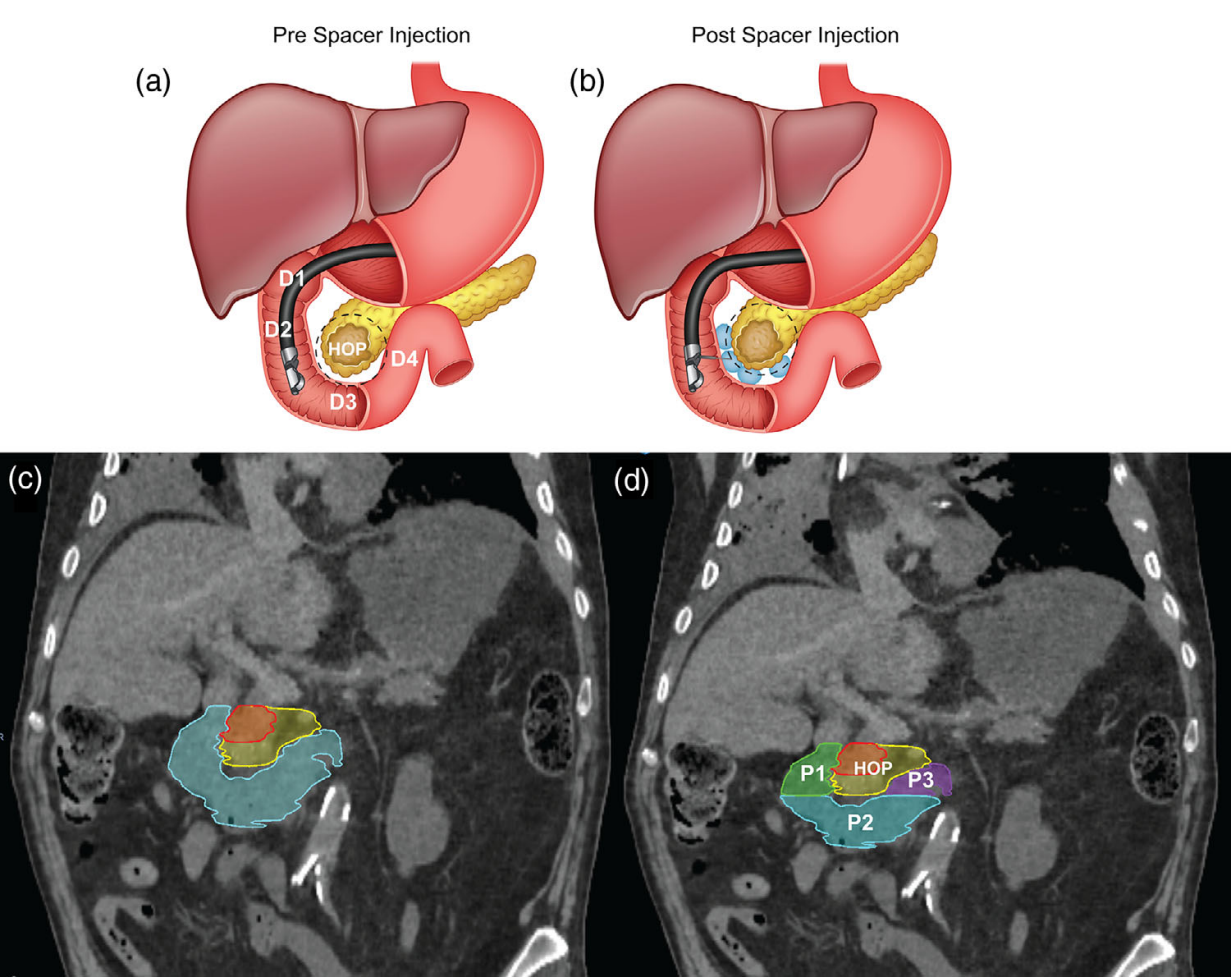
(A) Anatomically, the “C”‐shaped duodenum curves around the head of the pancreas (HOP) and is divided into four sections D1–D4. (B) The duodenal spacer placement increases the separation between the duodenum and pancreas. (C) Illustration of two GTV types and duodenal loop: The small (red) and large (yellow) GTV, along with the duodenal C loop. (D) The duodenal loop is divided into three main parts: descending (green), horizontal (cyan), and ascending (purple)

To ensure a well‐posed FE (Finite Element) problem, we used anatomic boundary conditions, inspired by the duodenum‐pancreas interface. Shown in Figure 1, anatomically, the duodenum is divided into four sections: (D1) the superior part of the duodenum, begins as a continuation of the pylorus, (D2) the descending section that begins at the D1 flexure, (D3) the horizontal section of the duodenum that begins at the inferior D2 flexure and passes transversely to the left, (D4) the ascending part of the duodenum that passes superiorly and ends at duodenojejunal flexure.^33^

We compared the pre‐ and postinjection scans to understand the effect of spacer placement. We observed that the inferior surface of the duodenal horizontal section (D3) relatively stays in the same position. However, the duodenal descending and ascending parts (D2 and D4) move considerably. Because of the stomach and sphincter higher stiffness, the movement of the duodenal section immediately after stomach (D1) is limited. We incorporated these anatomical restrictions by bounding the mesh nodes of the inferior surface of the D3 and the nodes within a 2‐mm distance from the stomach. Based on our observations, the HOP showed a local deformation, rather than a global movement. Thus, the superior and inferior margins of HOP mesh were fixated, preventing the target structure from global movement while allowing local deformation.

The model was validated on the postinjection scans from cadavers. For the validation purpose, the distribution of spacer was determined by aligning the pre‐ and postinjection scans by HOP, because of its lack of global movement as mentioned earlier. Three figures of merit were used for validation: the dice similarity coefficient, the radial nearest neighbor distance, and overlapped volume histogram (OVH).^24^ The node's transitions were converted to diffeomorphic deformation vector fields using our in‐house algorithm. Next, the deformation field was applied to the preinjection scan and structure set to create the postsimulation scan and structures. The nodes translation of the FE model was calculated using the ABAQUS commercial software package. The analysis was done on a Dell XPS 15, 7590, equipped with 2.4 GHz Intel Core i9, and 32 Gigabytes RAM.

### 2.3 Simulation of hydrogel spacer uncertainties

To perform the hydrogel spacer placement simulation, we divided the duodenum into three anatomical parts: P1, the descending part of the duodenum (D1 & D2); P2, the horizontal part (D3); P3, the ascending part (D4) (Figure 1D). We simulated two tumor sizes (small and large), based on the relative geometry of the HOP and duodenal loop. The size of the tumor determined which parts of the duodenum should be injected with the spacer. As seen in Figure 1C, for the small gross tumor volume (GTV) due to its proximity to descending part of the duodenum (P1), injection in P1 would be sufficient to spare the duodenum. On the other hand, the large GTV had an interface with the full duodenal C‐loop. Therefore, to better spare the duodenum, the spacer should be injected in the full duodenal C‐loop.

A single value 3D measurement using the OVH distance L1cc (tumor expansion overlapping 1cc duodenal volume) was used for the initial evaluation of the separation between OAR and tumor. The OVH is an on‐demand quantity that shows the 3D relative geometry of ROIs. Previously, it is shown to have a high correlation with dosimetric indices,^34^, ^35^ and used for automatic or semiautomatic treatment planning.^36^, ^37^, ^38^, ^39^ Our preliminary study showed that L1cc > 14 mm results in V33Gy that is close to zero (the duodenal volume receiving 33 Gy).^24^

To determine the optimal spacer distribution, we manually placed the blebs where the planning target volume (PTV) overlaps with the duodenum. In this study, our goal was to achieve 95% PTV volume coverage with prescription dose without violating OAR constraints. As a result, the chosen spacer distribution aims at minimizing the PTV overlapping volume with the duodenum.

**FIGURE 2:**
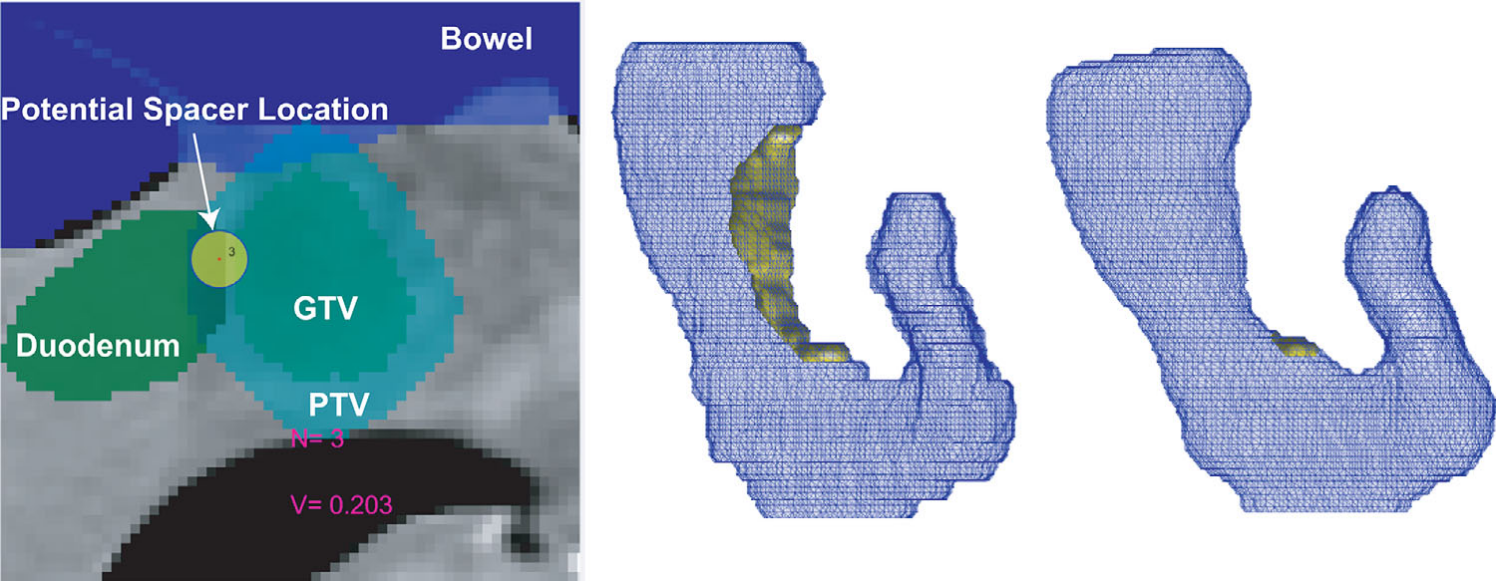
Spacer distribution was determined using the designed graphical user interface that utilizes the 2D visualization of ROIs. The figure also shows the representation of FEMOSSA user interface. N indicates the number of blebs currently placed. V shows the approximate volume of spacer so far. The yellow disk represents the bleb in 2D and the 3 shows the radius for the current bleb (A). The spacer distribution was chosen so that the PTV has minimum to no overlap with the duodenum. (B) The 3D visualization of ROIs and the PTV overlap with the duodenum (yellow volume). (C) The result of virtual spacer planning shows PTV has minimum overlap with the duodenum

Shown in Figure 2A, FEMOSSA's built‐in user interface allows choosing the location of the spacer (yellow disk) so that the overlapping volume of duodenum and PTV is minimized. Figure 2B shows the 3D visualization of preinjection PTV, with a large overlapping volume with the duodenum (the yellow shaded volume). The overlapping volume is considerably reduced after the placement of the ideal spacer distribution (Figure 2C).

For this feasibility study, two common uncertainties associated with spacer injection were simulated to show the corrective feedback: (1) narrowing uncertainty for the small GTV case and (2) missing part uncertainty for the large GTV case. The “narrowing” was defined as the injection of less volume of spacer as was suggested by the ideal injection scenario. To simulate the narrowing, we randomly reduced the radii of the blebs, resulting in a decrease in the overall volume of the spacer. The “missing part” was simulated by missing the injection in the ascending section of the duodenum (P3). Although the preoperative placement planning recommends the injection in P3, due to the hard‐to‐reach location of P3 it may not be injected.

### 2.4 Radiation therapy planning

For each case, the eight scenarios were planned with Stereotactic Body Radiation Therapy (SBRT) techniques. Each case has two GTV types with their corresponding uncertainties. For each GTV type, there are four scenarios: preinjection, ideal injection, nonideal injection, and corrective injection. Here, we used the 2D PTV overlap with the duodenum metric to determine the ideal distribution of the virtual spacer. The PTV was created based on the clinical planning protocol in our institute by first expanding GTV by 3 mm to get the mock multiple breath‐hold GTV (GTV‐multabc) and then expanded further by 2 mm.

**FIGURE 3:**
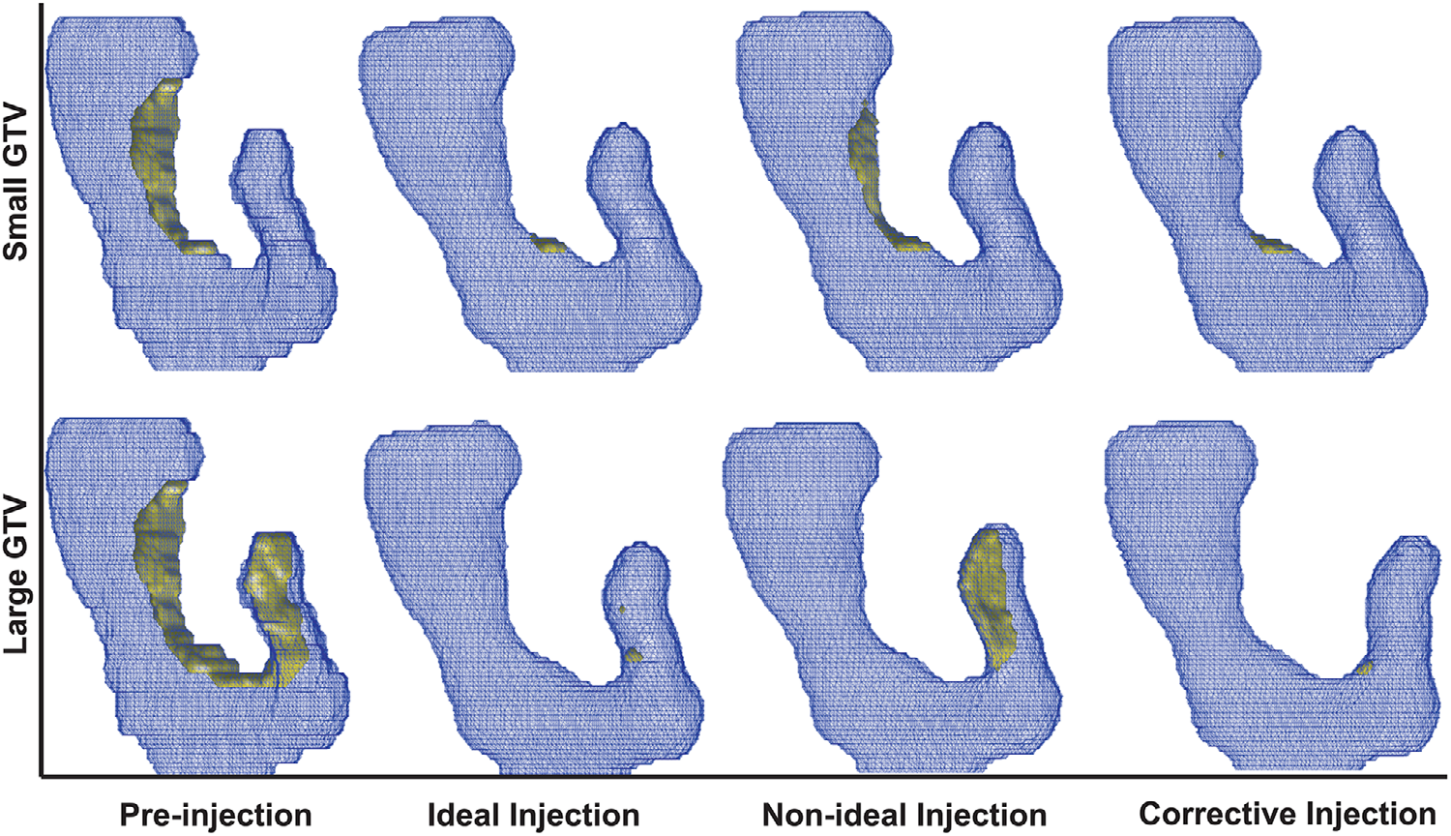
The 3D visualization of four stages of ROI. As seen, the Ideal Injection aims for removing the overlap between the PTV and duodenum (yellow volume). However, there are uncertainties associated with the procedure, narrowing (top row) and missing parts (bottom row). Using the intraoperative feedback, the corrective injection will be planned to remove the remaining overlapping volume

The preinjection scenario is based on the ROIs relative geometry before injection of the hydrogel. The ideal injection scenario is the simulation of the virtual spacer with the preoperative ideal spacer distribution. The nonideal injection scenario was simulated based on the distribution from the ideal scenario while incorporating the uncertainties. Finally, for the corrective injection case, the simulated ROIs from the nonideal case were used to perform another placement of the virtual spacer so that the PTV overlapping volume with the duodenum is minimized. Figure 3 shows an example case of the eight scenarios.

A total of 32 volumetric modulated arc therapy (VMAT) SBRT plans (33 Gy in 5 fractions) were designed, for four cadavers and eight scenarios. The planning objectives and constraints, approved by our institute board, were as follows: at least 95% of PTV volume receives ≥33 Gy, 100% of PTV volume receives ≥25 Gy, less than 1 cc of PTV volume receives ≥42.9 Gy, at least 95% of GTV‐multabc volume receives ≥33 Gy, 100% of GTV volume receives ≥33 Gy, less than 25% of kidney volume receives ≥12 Gy, less than 50% of liver volume receives ≥12 Gy, less than 20 cc of duodenum, stomach, and bowel volume receives ≥20 Gy, less than 1 cc of duodenum, stomach, and bowel volume receives ≥33 Gy, and less than 1 cc of spinal cord volume receives ≥8 Gy. To avoid any planning bias, the planning parameters, namely the number of beams, number of iterations, and objective functions were identical for all the plans. To make the plans comparable, later in optimization, we forced the optimization to achieve 95% PTV volume coverage by adding an extra constraint. The plans were designed and optimized using the RayStation treatment planning system (RaySearch Laboratories, Stockholm, Sweden).

### 2.5 Quantitative evaluation of procedure with corrective feedback

The ideal spacer distribution was chosen so that the PTV overlapping volume with the duodenum is minimized. As seen in the second column of Figure 3, this results in a small to no PTV‐duodenum overlap. However, the uncertainties during the procedure, namely narrowing and missing part, resulted in the nonideal distribution of spacer. A subsequent corrective injection was simulated by using the ROIs from the nonideal injection. The PTV overlapping volume with the duodenum is, again, used to determine the corrective spacer distribution. The simulated ROIs after each virtual injection were used for quantified comparison of the scenarios. First, we compared the 3D relative geometry of GTV and duodenum, and the distance between the two structures using the L1cc OVH distance. Second, the RT plans were compared in terms of the duodenal high dose volume, V33Gy, defined as the duodenal volume receiving the 33 Gy (prescribed dose).

## 3 RESULTS

**FIGURE 4:**
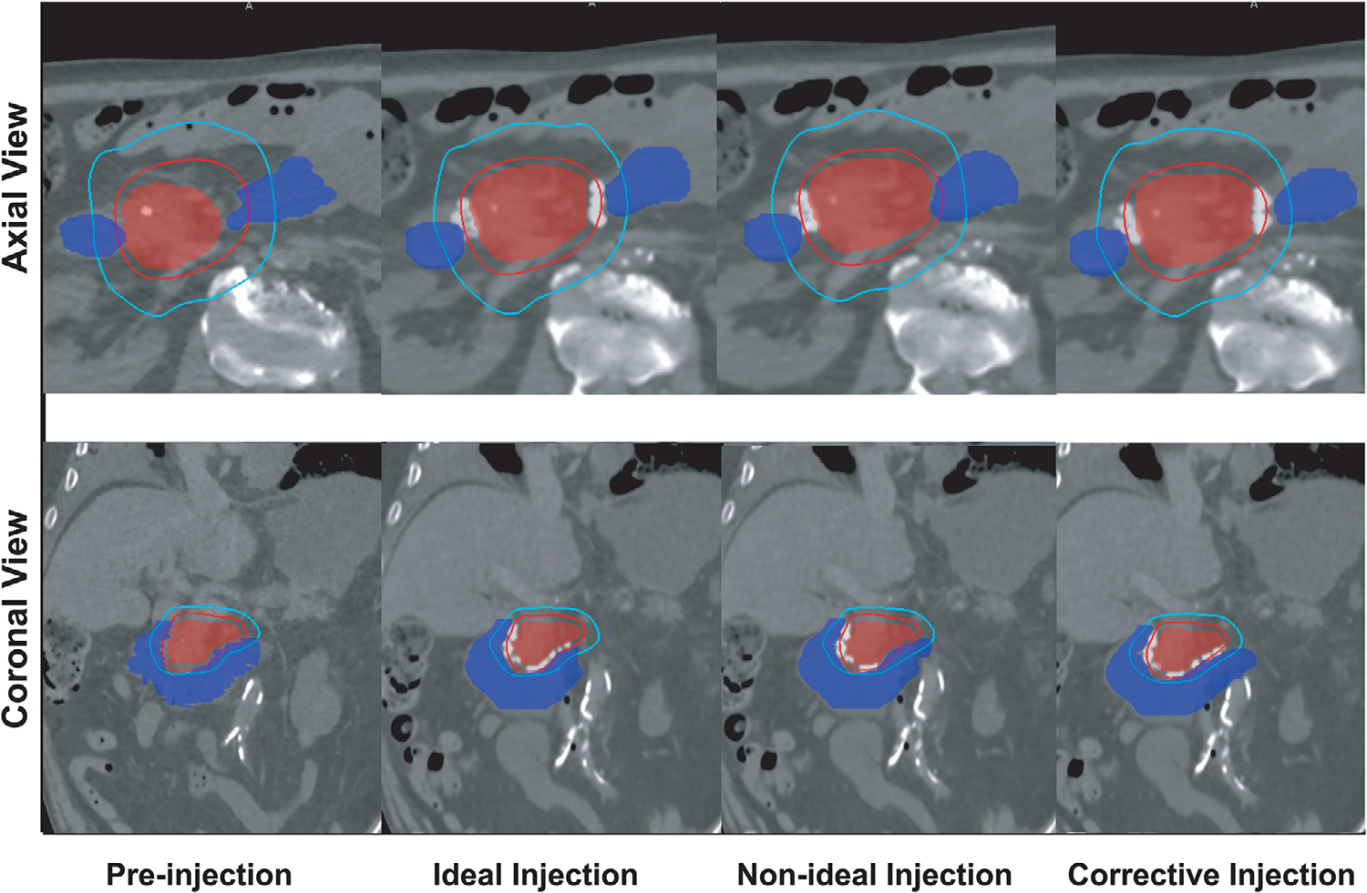
Illustration of 33 Gy (red) and 20 Gy (cyan) isodose line along with duodenum (filled blue) and GTV (filled red) for an example case. The top row shows the conventional axial view and the bottom row the coronal view which gives a better view of the duodenal loop and GTV

The preinjection GTV and duodenum contours were deformed using the simulated deformation vector field to create the postsimulation contours. The preinjection and deformed postsimulation contours were then used for RT planning and analysis. Figure 4 shows an example of SBRT planning for the large GTV case and all four scenarios (pre‐, ideal, nonideal, and corrective injection), alongside the 33 Gy (red) and 20 Gy (cyan) iso‐dose lines. As seen, although ideal injection of spacer resulted in almost completely sparing duodenum from 33 Gy dose cloud as expected, the nonideal injection of spacer resulted in the ascending part of duodenum being exposed to high dose radiation. The designed corrective injection was able to spare the missing part of the duodenum.

**FIGURE 5:**
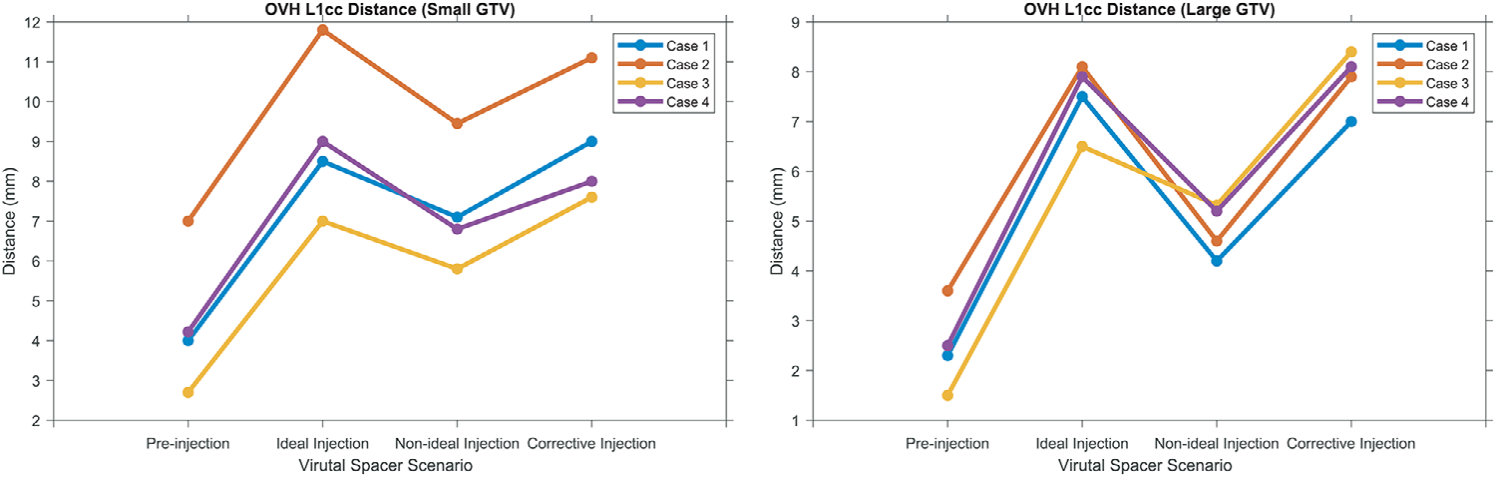
The quantified measurement and comparison of separation of OAR and target for all four scenarios and cases and two GTV types, small (A) and large (B). The separation is quantified by using L1cc distance

The spacer‐induced separation was measured using OVH L1cc distance. As seen in Figure 5, the ideal injection resulted in a noticeable increase in L1cc by an average of 5.3 and 4.6 mm for small and large GTV, respectively, reflecting the increase in separation between the OAR and target. For the nonideal injection scenario, the separation was less than what was originally planned. Finally, the corrective injection increases the separation close to the planned value. We believe the 3D OVH distance is an informative metric for 3D RT planning. Nonetheless, we have also reported the averaged 2D GTV‐duodenum distance in Supporting information (Tables S1, S2).

We also compared the duodenal high dose volume, V33Gy, as it is critical for dose escalation which is the key to the increase in OS rate. Although we used the same planning parameters depending on the patient anatomy to spare the duodenum, each plan may achieve a different amount of target coverage. As mentioned in the Section 2, we made the plans comparable by adding an extra constraint to force the optimization to achieve 95% PTV coverage. This constraint resulted in all the plans having a PTV 33 Gy coverage of between 95 and 96%.

**FIGURE 6:**
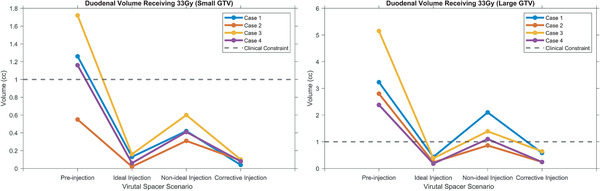
The V33Gy, duodenal volume receiving 33 Gy, for four scenarios of injection and all four cases, for (A) small GTV and (B) large GTV case. As seen, in both cases, the ideal injection resulted in high duodenal sparing, but the duodenum and nonideal injection reduced the efficacy of spacer, and finally, the corrective injection compensated for the uncertainties

The averaged preinjection duodenal V33Gy were 3.3 and 1.2cc for small and large GTV, respectively, and were reduced to 0.3 and 0.1cc after ideal spacer injection. Shown in Figure 6, compared to the ideal injection, the nonideal spacer injection led to less reduction in the duodenal V33Gy (on average, 1.3 and 0.4cc). The corrective injection further decreased the duodenal V33Gy and, therefore, compensated for the shortcomings of the nonideal injection to achieve averaged duodenal V33Gy of 0.4cc for large GTV and 0.1cc for small GTV.

## 4 DISCUSSION

In this work, we investigated the feasibility and advantages of the duodenal hydrogel spacer placement procedure featuring corrective feedback. The data from four cadavers were simulated with a virtual spacer using our in‐house, physics‐based, patient‐specific spacer simulation algorithm, FEMOSSA. Previously, we have applied FEMOSSA to rectal spacer and shown its advantages over previous models.^23^ Recently, we have applied FEMOSSA to duodenal spacer and shown that FEMOSSA can be used for simulating different scenarios of spacer placement to get a better insight into various aspects of duodenal hydrogel spacer^24^, ^40^ and potentially other sites.^41^ All these studies demonstrate the versatility of FEMOSSA and its potential to be used for other hydrogel spacer‐related applications such as drug delivery using hydrogels,^42^ biomaterial delivery,^43^ and plastic surgery.^44^ Moreover, FEMOSSA can be used to simulate numerous patient‐specific and diverse data that are useful to train artificial intelligence models that in turn can elevate the quality of spacer placement procedure.

To show the efficacy of the corrective feedback in the hydrogel spacer injection procedure, we used the OVH L1cc distance and the duodenal high dose volume (V33Gy). OVH is a useful 3D physical feedback measure that quantifies the spatial separation between target and OAR. OVH distances have been shown to be correlated with dosimetric indices, and have been used for predicting the duodenal dose, and automated and semiautomatic treatment planning.^35^, ^36^, ^37^, ^38^, ^39^ Here, we used OVH L1cc to measure the spacer‐induced separation. The choice of L1cc is because the duodenal V33Gy < 1cc constraint is the major limiting constraint in dose‐escalated pancreatic RT. Our results indicate that the OVH is sensitive to spacer distribution. Figure 5 shows that for both small and large GTV, the OVH L1cc follows the same pattern. First, the L1cc increased to the highest value with the injection of the ideal spacer distribution. Next, the uncertainties resulted in reducing the spacer‐induced separation, and thus, L1cc decreased considerably. Finally, the corrective injection increased the L1cc.

Because we performed two sequential simulations for the corrective injection scenario, the spacer distribution created for the ideal injection is different from that for the corrective injection. More specifically, first, the nonideal distribution of spacer was simulated, and then, the new scan and structures were used to determine the distribution of spacer needed to be injected. Then, we performed a second FE simulation to create the corrective injection. We believe that this method results in a more realistic simulation of corrective injection. Thus, as expected, the nonlinearity of the virtual spacer simulation resulted in getting different values for the corrective injection than the ideal injection, which more closely resembles what happens in the actual injection procedure.

To find the ideal location of the spacer, we proposed using the overlapping PTV volume with the duodenum. In practice, the PTV volume is used to incorporate uncertainties, like motion and setup uncertainty. Therefore, minimizing the duodenum‐PTV overlap not only increases the duodenum sparing, but also can introduce the spacer as a buffer‐like structure to reduce RT planning uncertainties.^45^ Moreover, we found that although dosimetric and complex anatomical feedback, like OVH, are useful for optimizing the preoperative location of the spacer, these feedbacks may not be as informative as PTV overlap with OAR to a gastroenterologist.

The duodenum‐PTV overlapping volume can also provide informative feedback during the procedure. Because the procedure is done using an ultrasound endoscopic probe, the gastroenterologist can only inspect the outcome of injection from the 2D ultrasound scan. Thus, the 2D measurement of the distance between tumor and duodenum is useful guidance for the procedure. However, the main challenge is the registration of 2D ultrasound images to the CT and measuring the amount of separation, which is the aim of our current and future studies.^46^, ^47^, ^48^

We are aware that our study has a few limitations. First, due to the novelty of the duodenal spacer, the number of cases is very limited and has not been widely used in the clinic. One potential reason may be the complexity and the high uncertainty in the actual outcome of the procedure, which is the main motivation of this work. Thus, we believe that this feasibility study shows that using corrective feedback improves the spacer procedure outcome and promotes the use of the spacer to improve the quality of RT treatment.

Moreover, here we did not incorporate the effect of breathing and normal organ movements, as these movements may induce further uncertainties for the actual procedure. The main goal of this study, however, was to demonstrate the effectiveness of the preoperative design, intraoperative evaluation, and potential correction. Although unavoidable, by taking advantage of the near real‐time AI‐based systems, we believe the effect of this uncertainty can be minimized. Currently, we are developing a portable C‐arm X‐ray‐based AI (Artificial Intelligence) feedback system, where the X‐ray images are intraoperatively acquired to locate and track the spacer. The spacer is automatically segmented and then its volume is reconstructed from the X‐ray projections in near real‐time to be compared with the ideal distribution of spacer. As a result, this system can provide the physician with comprehensive image guidance for potential corrective injections.

Another limitation of the study is that we only considered two types of uncertainties in the spacer placement procedure. However, there are more underlying uncertainties involved in the process, for instance, the uncertainty in the FE modeling process, like computing platform, choice of boundary conditions, element type, and material properties.^49^ We tried to minimize these uncertainties, by validating FEMOSSA on pre‐ and postinjection pair scans of the cadavers. Moreover, there are other uncertainties that need to be addressed, for instance, the optimal location of the spacer spacer and how much hydrogel volume is needed. Previously, our group has shown that small hydrogel volume (<5cc) only creates a small separation (<2 mm).^3^, ^8^ In more recent clinical trials in our institute, a larger hydrogel volume was injected, and although it resulted in a lower duodenal dose, our simulation study showed that the spacer distribution was not the optimal location for achieving the maximum benefit.^8^, ^24^ Accordingly, further studies are underway by our group to first predict the optimal location of the spacer and implement the intraoperative feedback to guide the procedure.

## 5 CONCLUSIONS

In this work, we investigated the feasibility and benefits of intraoperative corrective feedback for the duodenal spacer placement procedure. Our simulation result showed that corrective feedback compensated for common uncertainties associated with the spacer placement procedure, and thus, increased the effectiveness of the complicated EUS‐guided spacer placement. We showed that PTV overlapping volume with OAR is on‐demand and informative potential intraoperative feedback that can guide the physician during the procedure. Future work focuses on (1) developing a decision support system that predicts the optimum location of the spacer, (2) implementing the intraoperative feedback system to localize the spacer and provide quantitative and visual feedback during the actual procedure.

## CONFLICT OF INTEREST

The authors have no conflict to disclose.

## Supporting information

Supporting information

Supporting information

## Data Availability

The raw data supporting the conclusions of this article will be made available by the authors upon request.
