# Supplementary material for: Demonstrating the benefits of corrective intraoperative feedback in improving the quality of duodenal hydrogel spacer placement
Source: Med Phys. 2022 Apr 18;49(7):4794–803. doi: 10.1002/mp.15665 (PMC9540875; doi:10.1002/mp.15665)
Supplement: Supplementary file 1 — Supporting information [file MP-49-4794-s001.pdf]

Table S-1: Averaged 2D distance (mm) between GTV and duodenum for small GTV scenario and section 1 (S1)

Case Number	Pre-injection	Ideal Injection	Non-ideal Injection	Corrective Injection
Case 1	1.6	6.3	4.6	6.5
Case 2	4.2	7.1	5.2	6.9
Case 3	0.6	6.6	4.3	6.7
Case 4	1.5	6.9	3.7	5.6

Please note measurement was done on coronal view where the spacer is best visualized.
