# Supplementary material for: Demonstrating the benefits of corrective intraoperative feedback in improving the quality of duodenal hydrogel spacer placement
Source: Med Phys. 2022 Apr 18;49(7):4794–803. doi: 10.1002/mp.15665 (PMC9540875; doi:10.1002/mp.15665)
Supplement: Supplementary file 2 — Supporting information [file MP-49-4794-s002.pdf]

Table S-2: Averaged 2D distance (mm) between GTV and duodenum for large GTV scenario and for all sections (S1, S2, and S3)

Case Number	Pre-injection			Ideal Injection			Non-ideal Injection			Corrective Injection		
Section	S1	S2	S3	S1	S2	S3	S1	S2	S3	S1	S2	S3
Case 1	1.4	3.8	1.7	7.2	5.6	6.2	7.0	5.1	1.6	7.4	5.3	5.3
Case 2	4.2	5.7	3.8	6.0	5.8	5.8	6.2	5.8	4.4	6.4	5.7	5.6
Case 3	2.5	1.7	1.1	5.2	6.4	7.0	5.3	6.5	3.1	5.3	6.8	7.4
Case 4	1.4	4.0	1.7	7.3	5.1	6.3	7.1	4.8	3.0	7.4	5.6	6.9

Please note measurement was done on coronal view where the spacer is best visualized.
